# A multi-site laboratory evaluation of the MEDSCAN application for automated POC-CCA interpretation

**DOI:** 10.3389/fpara.2026.1837310

**Published:** 2026-06-19

**Authors:** Thomas F. Scherr, Pytsje T. Hoekstra, Theresia Abdoel, Carson P. Moore, Austin Hardcastle, Matthew Boisse, Boaz Mulonga, Kennedy Andiego, George Ogara, Gladys Odhiambo, Fredrick Rawago, Nicholas Spurlock, Alexandrea A. Howard, Megan E. Pask, Eline M. van Dam, Govert J. van Dam, Chris Ho, Goylette F. Chami, Frederick Haselton, Maurice R. Odiere, David Wright

**Affiliations:** 1 Department of Chemistry, Vanderbilt University, Nashville, TN, United States; 2 Leiden University Center for Infectious Diseases, Leiden University Medical Center, Leiden, Netherlands; 3 Mondial Diagnostics, Amsterdam, Netherlands; 4 Centre for Global Health Research, Kenya Medical Research Institute, Kisumu, Kenya; 5 Department of Biomedical Engineering, Vanderbilt University, Nashville, TN, United States; 6 Big Data Institute, Nuffield Department of Population Health, University of Oxford, Oxford, United Kingdom

**Keywords:** schistosomiasis, lateral flow assay, POC-CCA, disease surveillance, smartphone-based diagnostics, mobile health (mHealth), digital health, computer vision

## Abstract

**Introduction:**

Control efforts against schistosomiasis are hampered by the subjective interpretation of the point-of-care circulating cathodic antigen (POC-CCA) urine test, which limits diagnostic consistency. We developed MEDSCAN (Mobile-Enabled Diagnostics for Schistosomiasis Control Analytics), a mobile application that uses smartphone imaging and computer vision to automate POC-CCA interpretation.

**Methods:**

In a multi-site laboratory evaluation across the USA, the Netherlands, and Kenya, we compared MEDSCAN to visual G-Score interpretation and a benchtop lateral flow reader (LFR).

**Results:**

All three methods produced clear concentration–response relationships, with normalized machine-based metrics achieving AUROC ≥ 0.90. MEDSCAN demonstrated excellent inter-user reproducibility (intra-class correlation coefficients exceeding 0.94) and substantial agreement with both visual and LFR interpretations across sites. Some device-to-device variability was observed, attributable to differences in smartphone camera hardware and image processing; however, binary diagnostic outcomes remained robust across a heterogeneous set of smartphones.

**Discussion:**

These results establish operational positivity thresholds for MEDSCAN—based on test-line signal alone or normalized metrics—suitable for direct implementation in field surveillance workflows. A large-scale field study is underway to evaluate MEDSCAN under routine POC-CCA surveillance conditions in endemic settings.

## Introduction

Schistosomiasis is a neglected tropical disease that affects over 200 million people worldwide, primarily in regions with poor sanitation and limited access to clean water (McManus et al., 2018; Buonfrate et al., 2025). The disease is caused by parasitic worms of the genus *Schistosoma* and is transmitted through contact with contaminated freshwater (Brooker et al., 2009). Chronic infections can lead to severe morbidity, including anemia, organ damage, and increased susceptibility to other infections, contributing to significant public health and socioeconomic burdens (King et al., 2005; Gryseels et al., 2006; Butler et al., 2012; Colley and Secor, 2014). Despite extensive efforts to control the disease, schistosomiasis remains endemic in many areas, particularly in sub‐Saharan Africa (Adenowo et al., 2015).

One of the major challenges in schistosomiasis control is the presence of persistent transmission hotspots—areas where infection rates remain high despite repeated mass drug administration (MDA) campaigns (Kittur et al., 2019; Kittur et al., 2020). These persistent hotspots can be attributed to multiple factors, including reinfection from environmental reservoirs, limitations in MDA coverage, an imperfect cure rate from Praziquantel, and gaps in traditional surveillance methods (Colley, 2014; French et al., 2018; Secor and Colley, 2018; Amoah et al., 2020). Scalable diagnostic tools are needed to monitor infections within persistent hotspots and, as programs shift from control toward elimination, to detect low-intensity infections that sustain transmission (WHO guideline on control and elimination of human schistosomiasis, 2022). Building on these observations, the World Health Organization (WHO) and other public health bodies have emphasized the need for improved surveillance strategies to accelerate progress toward schistosomiasis elimination (Ending the neglect to attain the Sustainable Development Goals: A road map for neglected tropical diseases 2021–2030; Secor and Colley, 2018; WHO guideline on control and elimination of human schistosomiasis, 2022). High-resolution surveillance requires not only reliable diagnostic methods but also real-time data capture, which enables rapid aggregation and spatial mapping of results to guide more responsive control strategies (Tchuem Tchuenté et al., 2017; Tchuem Tchuenté et al., 2018).

The point-of-care circulating cathodic antigen (POC-CCA) urine test has become a widely used diagnostic tool for *Schistosoma mansoni*, offering a non-invasive, field-deployable alternative to stool-based Kato-Katz microscopy (Colley et al., 2013; Bärenbold et al., 2018; Hoekstra et al., 2021). Its strengths include ease of use and higher sensitivity in low- and moderate-intensity infection settings, making it particularly valuable for surveillance (Colley et al., 2013). The POC-CCA is a lateral flow immunoassay in which a urine sample migrates across a nitrocellulose membrane housed in a plastic cassette, producing a colored test band when CCA is present. However, interpretation of POC-CCA results is observer-dependent and often complicated by faint or borderline bands (Colley et al., 2017; Peralta and Cavalcanti, 2018; Colley et al., 2020). Several approaches have sought to reduce this observer dependence, including our previous work on quality control in Uganda (Ho et al., 2026), semi-quantitative scoring systems (Casacuberta-Partal et al., 2019), digital image analysis in Tanzania (Casacuberta et al., 2016), dedicated strip readers in Cameroon (Mewamba et al., 2021), and assessments of interpretation variability in Brazil (Carvalho et al., 2024). These studies highlight both the feasibility and the persistent need for standardized interpretation methods for the POC-CCA. While each of these approaches addresses specific aspects of interpretation standardization, none integrates automated image-based interpretation with programmatic data capture in a field-deployable platform. More broadly, integration of diagnostic tools with digital health technologies offers a promising pathway to enhance diagnostic capacity and epidemiological monitoring (Wood et al., 2019; Greve et al., 2022).

**Figure 1:**
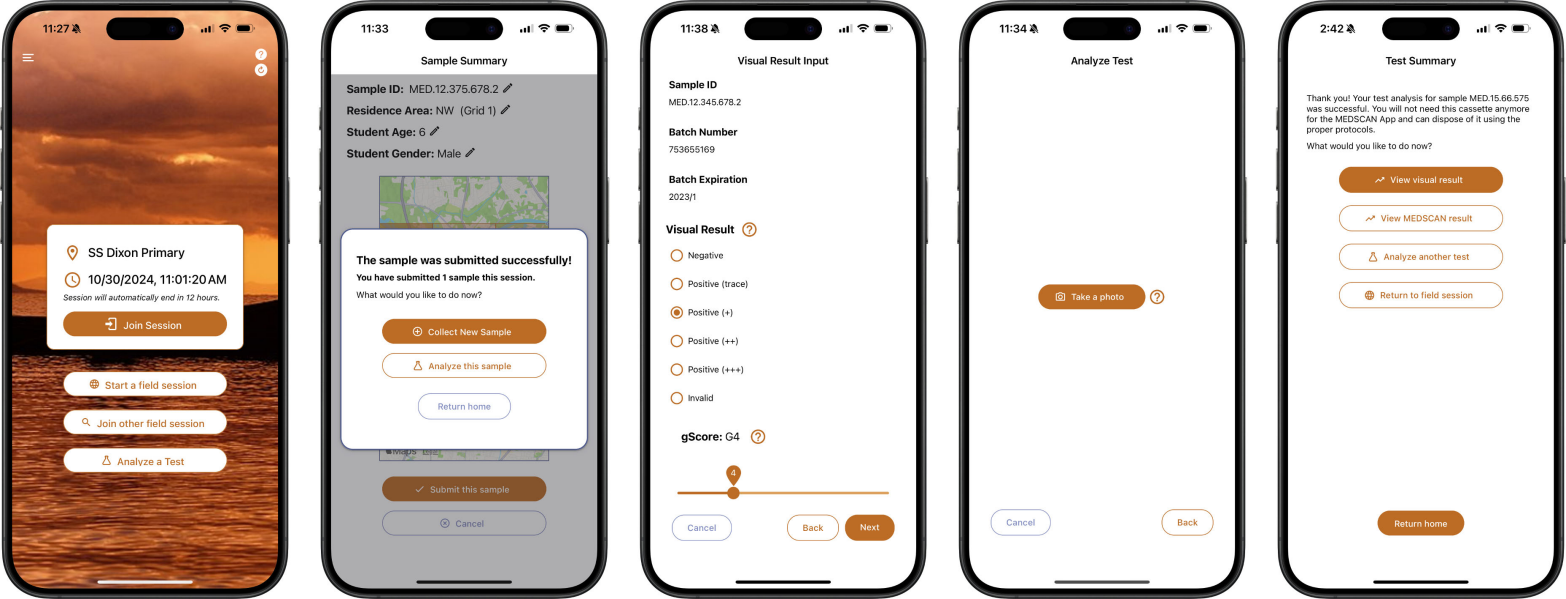
The MEDSCAN app for schistosomiasis surveillance and control. From left: a home screen after authentication showing available testing sessions to join; a summary screen at the conclusion of sample collection; a screen for inputting the visual test result; a screen to collect a photograph of the POC-CCA that will be automatically analyzed; a summary of MEDSCAN and visual results.

To address these needs, we developed MEDSCAN (Mobile-Enabled Diagnostics for Schistosomiasis Control Analytics), a mobile application built specifically for the POC-CCA test (**Figure 1**). MEDSCAN enables rapid, objective, and reproducible schistosomiasis diagnosis through automated image-based analysis, drawing on our prior experience developing mobile readers for malaria and HIV rapid diagnostic tests (Scherr et al., 2016; Moore et al., 2021; Schnall et al., 2025). By combining smartphone-based image interpretation with real-time, geo-tagged data capture, MEDSCAN provides standardized, reproducible results while also generating high-resolution epidemiological data. The application integrates user-friendly sample collection record-keeping with automated image processing in an interface designed for low-resource settings. It is intended to reduce reliance on expert diagnostic technicians while strengthening surveillance and control programs through scalable, interoperable digital reporting.

In this study, we evaluated the image-processing and interpretation components of the MEDSCAN platform using a dedicated MEDSCAN Lab application to establish practical, reproducible positive thresholds for direct field implementation. Specifically, we aimed to assess the agreement between MEDSCAN-derived interpretations and conventional visual scoring across multiple users and sites, to benchmark MEDSCAN outputs against a laboratory-based lateral flow reader, and to identify signal-based metrics and thresholds suitable for standardized implementation.

## Materials and methods

**Figure 2:**
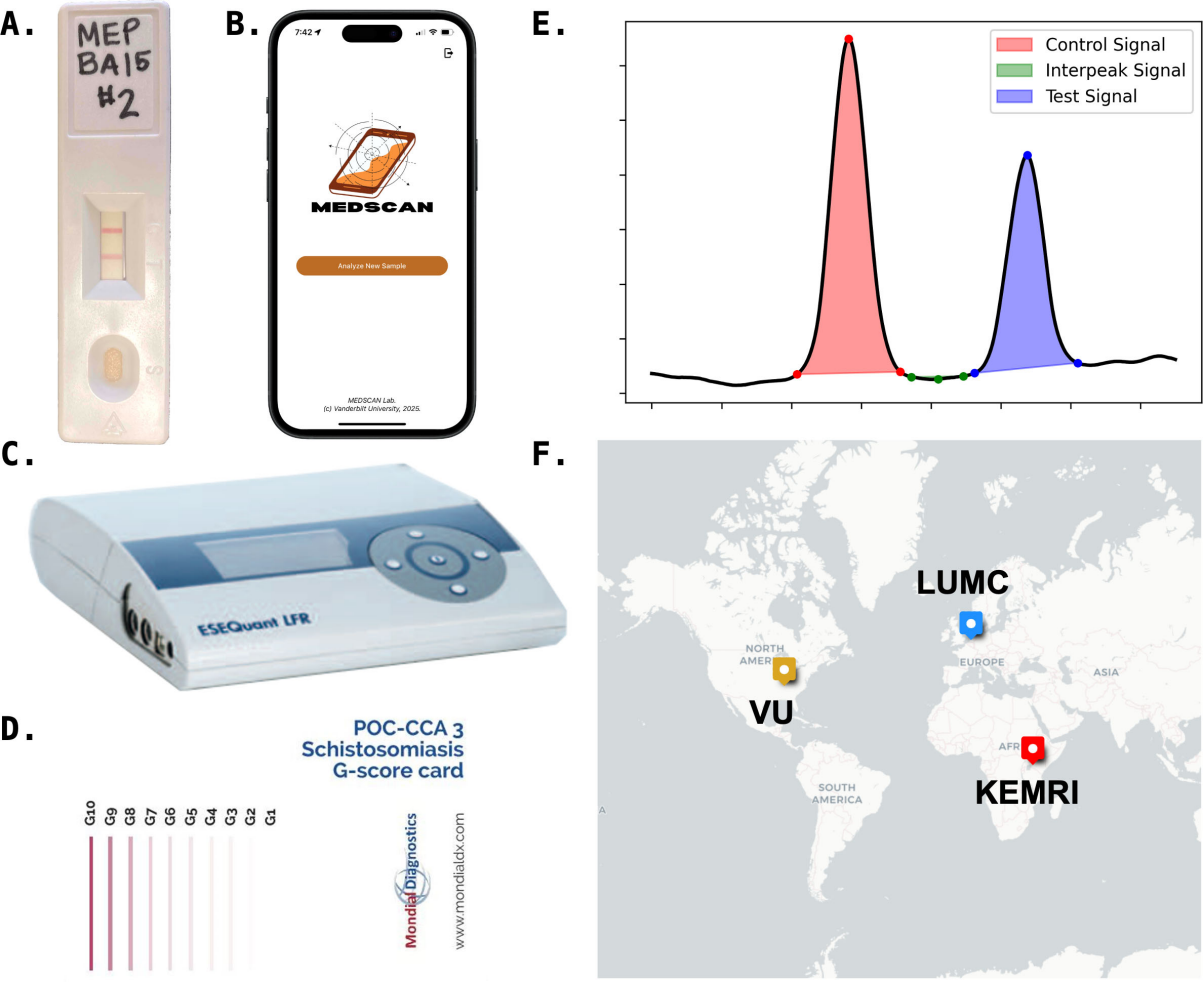
**(A)** Diagnostic test photo, **(B)** MEDSCAN Lab app, **(C)** LFR, **(D)** G-Score card, **(E)** Representative MEDSCAN linescan showing test signal, interpeak signal, and control signal, **(F)** Lab evaluation site map.

**Figure 3:**
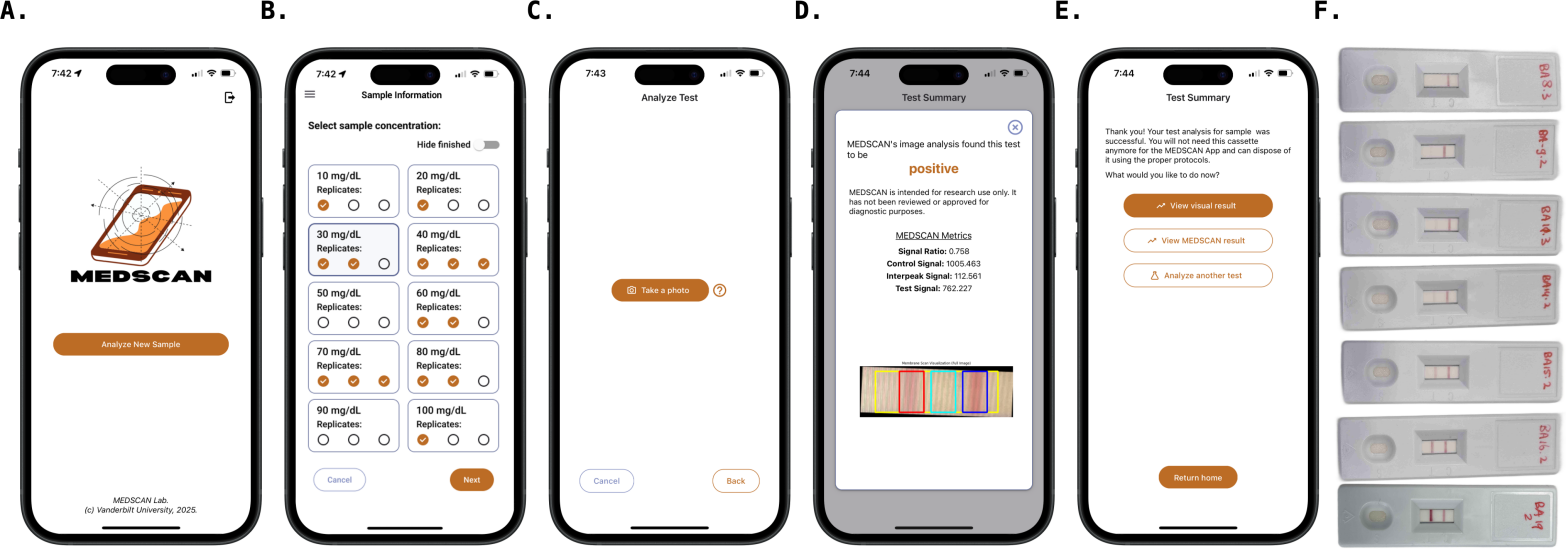
**(A–E)** Screenshots of MEDSCAN Lab app. **(F)** Representative photographs spanning the range of analyte concentrations tested.

For this study, we developed a dedicated MEDSCAN Lab app that isolates the image-processing and interpretation components of the full MEDSCAN platform (**Figure 2**). This stripped-down version excluded field-specific features (e.g., sample collection, data synchronization, and programmatic reporting) to enable a rigorous multi-site evaluation of algorithm performance and to establish practical positivity thresholds under controlled conditions (**Figure 3**). The thresholds derived here are intended for direct use in the full MEDSCAN field application.

### Laboratory testing sites

The laboratory evaluation was performed at three sites: Leiden University Medical Center (LUMC; Leiden, the Netherlands), the Centre for Global Health Research, Kenya Medical Research Institute (KEMRI; Kisumu, Kenya), and Vanderbilt University (VU; Nashville, USA). At each of the three sites, three experienced laboratory technicians performed all test analyses. At LUMC, KEMRI, and VU tests were analyzed by visual inspection and the MEDSCAN app. At VU, tests were additionally analyzed using a lateral flow reader (LFR).

### Sample preparation and use of POC-CCA diagnostic tests

Trichloroacetic acid-soluble fraction of *S. mansoni* adult worm antigen (AWA-TCA), containing approximately 3% (w/w) circulating cathodic antigen (CCA) (Van Dam et al., 1994), was used to spike CCA-negative human urine. Analyte-free human urine was spiked with the AWA-TCA ranging from 0 to 240 ng CCA/mL (0.3, 1, 1.5, 2.1, 2.4, 2.7, 3.3, 3.9, 4.5, 24 and 240 ng CCA/mL). The spiked urine samples were lyophilized and shipped to the testing laboratories for analysis. Prior to use, samples were rehydrated with deionized water.

The POC-CCA (version 3) was purchased from Mondial Diagnostics (batch number SCH3-24Apr2024, Amsterdam, the Netherlands). Tests were performed following manufacturer instructions. Briefly: using the exact volume pipette supplied in the kits, 100 μL of sample was pipetted to the sample well. After 20 minutes, test results were interpreted by visual inspection, the MEDSCAN Lab app, and LFR. All results interpretation methods were performed within the valid 10-minute test analysis window (20–30 minutes after sample addition to the cassette). Each concentration was analyzed in triplicate.

### Visual interpretation of test results

G-Scores have become a widely used approach to provide an improved semi-quantitative visual scoring of the POC-CCA (Casacuberta-Partal et al., 2019), which provides an aid to health workers and also creates a graded scale. This scale is useful because test line intensity is relative to the level of CCA in the sample, which has been shown to correlate with infection intensity at the population level (Kittur et al., 2016), while acknowledging that individual-level variability may occur. As such, each testing site was provided with a set of G-Score cassettes, and tests were analyzed by visual inspection using the semi-quantitative G-Score system. To streamline data collection, a screen within the MEDSCAN app was included to record the visual G-Score result for a particular sample. Visual scoring was not blinded to concentration; this is discussed further in the Limitations section.

### MEDSCAN Lab app

The MEDSCAN Lab app, which included only a subset of features from the full MEDSCAN platform, was distributed to study team members through Google Play Console and Apple TestFlight, depending on the device. This enabled evaluation across a heterogeneous set of smartphones differing in camera resolution, onboard image-processing pipelines, and display characteristics. Devices used in the study included Oppo A16K, CPH2349, Samsung SM-G991B and Galaxy M33, Google Pixel 4a and Pixel 7, and Apple iPhone 11 Pro, 13, and 14 Pro Max. To increase device parity during testing, users were not requested to use any particular device; as a result, phone models were not shared across sites (see Limitations).

Details of the computer-vision model and training procedure, as well as signals processing, are provided in the Supplemental Information. Cassette recognition performance was quantified using mean Average Precision (mAP) across Intersection-over-Union (IoU) thresholds, consistent with standard object-detection evaluation. To analyze a test, users photographed the POC-CCA cassette using the MEDSCAN Lab app’s built-in camera interface (**Figure 3**). The app’s image processing algorithm then automatically detected the cassette, identified the test membrane, and quantified test, control, and interpeak signal intensities from the captured image.

### Lateral flow reader analysis

The LFR, a benchtop instrument for laboratory evaluation of LF tests, was used to scan and quantify the POC-CCA lateral flow test strips. In addition to visual inspection and analysis by the MEDSCAN Lab app, at the VU site, immediately after analysis with the MEDSCAN Lab app, and still within the valid 10-minute test analysis window, each strip was removed intact from its cassette and analyzed using a Qiagen ESEQuant LFR (QIAGEN Lake Constance GmbH, Stokach, Germany), as described in previous work (Scherr et al., 2016; Moore et al., 2021). The linescan data (position in mm and signal in mV) was exported from the Qiagen software as a CSV, to allow for the evaluation of different metrics for positivity using custom Python software.

### Statistical analysis

All analyses were conducted using Python (v3.11.2) with the pandas, scikit-learn, statsmodels, and pingouin libraries. Data and code necessary to reproduce the findings presented in this manuscript are available at: https://doi.org/10.6084/m9.figshare.31838509.

#### Threshold determination and data binarization

For the MEDSCAN Lab app, several quantitative signal metrics were evaluated to characterize diagnostic performance: a) raw test-line signal, b) test-to-control signal ratio, c) test-to-interpeak ratio (background-corrected test signal), and d) the adjusted positivity index (API), a background-corrected test-to-control ratio given by Equation 1.

(1) $$Adj.\;Pos.\;Idx.\;=\;\frac{Test\;-\;Interpeak}{Control\;-\;Interpeak}$$

The API uses the interpeak signal as a background estimate; subtracting it from both the test and control signal intensities yields a background-corrected test-to-control ratio. The test-to-interpeak ratio is based on analytical chemistry approaches that define a detectable signal as 3× the standard deviation of the interpeak (background) signal.

For each of these metrics, as well as for the LFR test-line signal, receiver operating characteristic (ROC) curves were generated and Youden’s J statistic, Equation 2, was used to identify the threshold that maximized discrimination between positive and negative samples.

(2) J = Sensitivity + Specificity − 1

Samples with a concentration of ≥2.4 ng/mL were designated as positive, based on the detection threshold established by Polman et al. (2000) (Polman et al., 2000) using ELISA-based CCA detection. This threshold has been carried through multiple generations of the lateral flow assay, including the current POC-CCA3, using the same partly purified adult worm antigen preparation for calibration continuity. The value has occasionally been rounded to 2 ng/mL in the literature (van Dam et al., 2004).

For visual interpretation, a G-Score ≥ 4 was defined as positive. Using these ROC-derived thresholds, continuous outputs from the MEDSCAN Lab app and the LFR were converted into binary classifications (positive/negative).

#### Inter-user and intra-site variability

To assess variability among raters under identical test conditions, the intra-class correlation coefficient (ICC) was computed using a two-way random-effects model that estimates absolute agreement. Although each site performed the POC-CCA tests independently, each sample was prepared and run in triplicate by a single technician, and all replicates were subsequently interpreted independently by three users at that site. Data were structured so that each unique test condition (defined by a composite identifier derived from concentration and replicate number) served as the common target across users. Separate ICC analyses were performed for the MEDSCAN Lab app and visual G-Score outputs to quantify inter-user variability, and ICC was also computed within each site (i.e., intra-site variability) to determine whether systematic differences existed among sites. ICCs were used as the primary metric of inter-user and intra-site reliability.

#### Pairwise method agreement across methods, sites, and devices

To complement the ICC analyses, pairwise Cohen’s kappa statistics were calculated to assess agreement in binary diagnostic classifications at multiple levels: between methods, between sites, and between devices. For method-level comparisons, Cohen’s kappa was computed between MEDSCAN Lab app and visual G-Score classifications (obtained from the same user under identical conditions), as well as between the LFR and each of the other two methods for the subset of tests at VU where LFR data were available. For site- and device-level comparisons, experimental replicates were first aggregated by taking the mean for each group at each concentration; the aggregated values were binarized using the predetermined thresholds, and pairwise Cohen’s kappa was computed across all unique group pairs. Detailed user-level and device-level kappa values are reported in the Supporting Information.

#### Additional analyses

For continuous comparisons between methods, regression analyses and Bland–Altman plots were employed to characterize the relationship and agreement between the MEDSCAN Lab app and the LFR outputs across the full range of analyte concentrations. In a Bland–Altman plot, the y-axis represents the difference between the two methods (MEDSCAN Lab app minus LFR) while the x-axis represents the average of the two methods, allowing for the detection of proportional bias. A residual analysis was also performed, plotting the same differences against concentration to assess whether systematic biases change across the concentration range.

## Results

### Visual inspection results

**Figure 4:**
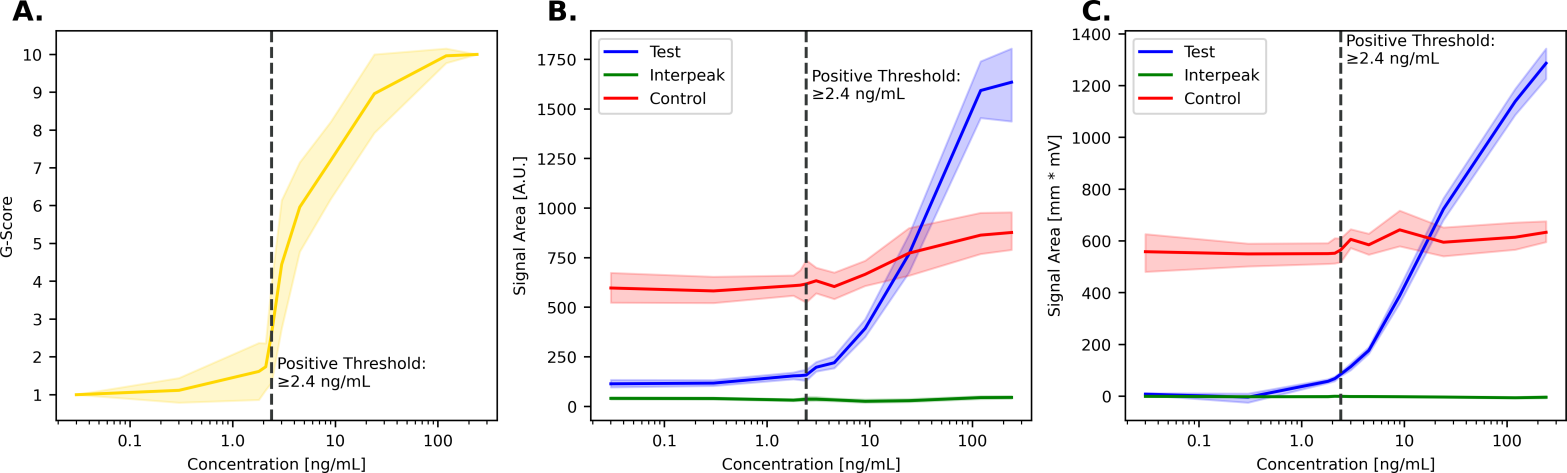
**(A)** Aggregated G-Scores across all users and replicates plotted against the concentration of spiked urine samples. The y-axis shows the full G-Score scale (1–10), reflecting the semi-quantitative scoring options available to users. Shaded regions indicate standard deviation. **(B)** MEDSCAN image processing test, interpeak, and control signals aggregated across all users and replicates. **(C)** Lateral flow reader test, interpeak, and control signals aggregated across all users and replicates. Shaded regions in **(B)** and **(C)** indicate 95% confidence intervals. For all, the dashed vertical line indicates the established positivity threshold (≥2.4 ng/mL).

The G-Score system, a common visual interpretation of test line intensity for the POC-CCA test, provides a semi-quantitative assessment based on human-perceived band intensity. Increasing G-Scores generally corresponded to higher concentration values (**Figure 4A**). Some variability was observed, particularly at lower concentrations around or below the threshold (2.4 ng/ml).

### MEDSCAN results

The MEDSCAN Lab app analyzed test strips using images captured via smartphone cameras. Control lines were detected in all images, and the app was able to correctly identify the location of the test line (present or not) in all images. Test and control line intensities were calculated across all concentrations, producing a dose-response trend (**Figure 4B**). Some variability between phone models was observed, particularly at lower concentrations. When stratified by site, G-Scores showed modest systematic differences: at concentrations just below 2.4 ng/mL, KEMRI’s G-Scores were slightly lower than the other sites, and at higher concentrations KEMRI had higher G-Scores than the other sites (**Supplementary Figure 1A**). MEDSCAN API results stratified by site (**Supplementary Figure 1B**) did not show any meaningful difference between sites.

### LFR results

The benchtop LFR system was used to quantify test line and control line intensities across different sample concentrations in the laboratory. LFR measurements showed a clear dose-response relationship, where increasing analyte concentration correlated with a higher test signal (**Figure 4C**). The upward trend of test signal started after 0.3 ng/mL, and clear differentiation between the background signal (interpeak area) was seen at the POC-CCA’s targeted positive threshold of 2.4 ng/mL. Minor deviations in linearity were observed at the higher concentrations, although a clear plateau was not seen. The control line remained relatively stable across all tests, and the interpeak signal remained consistently near zero.

### MEDSCAN metric-based thresholds and diagnostic performance

**Figure 5:**
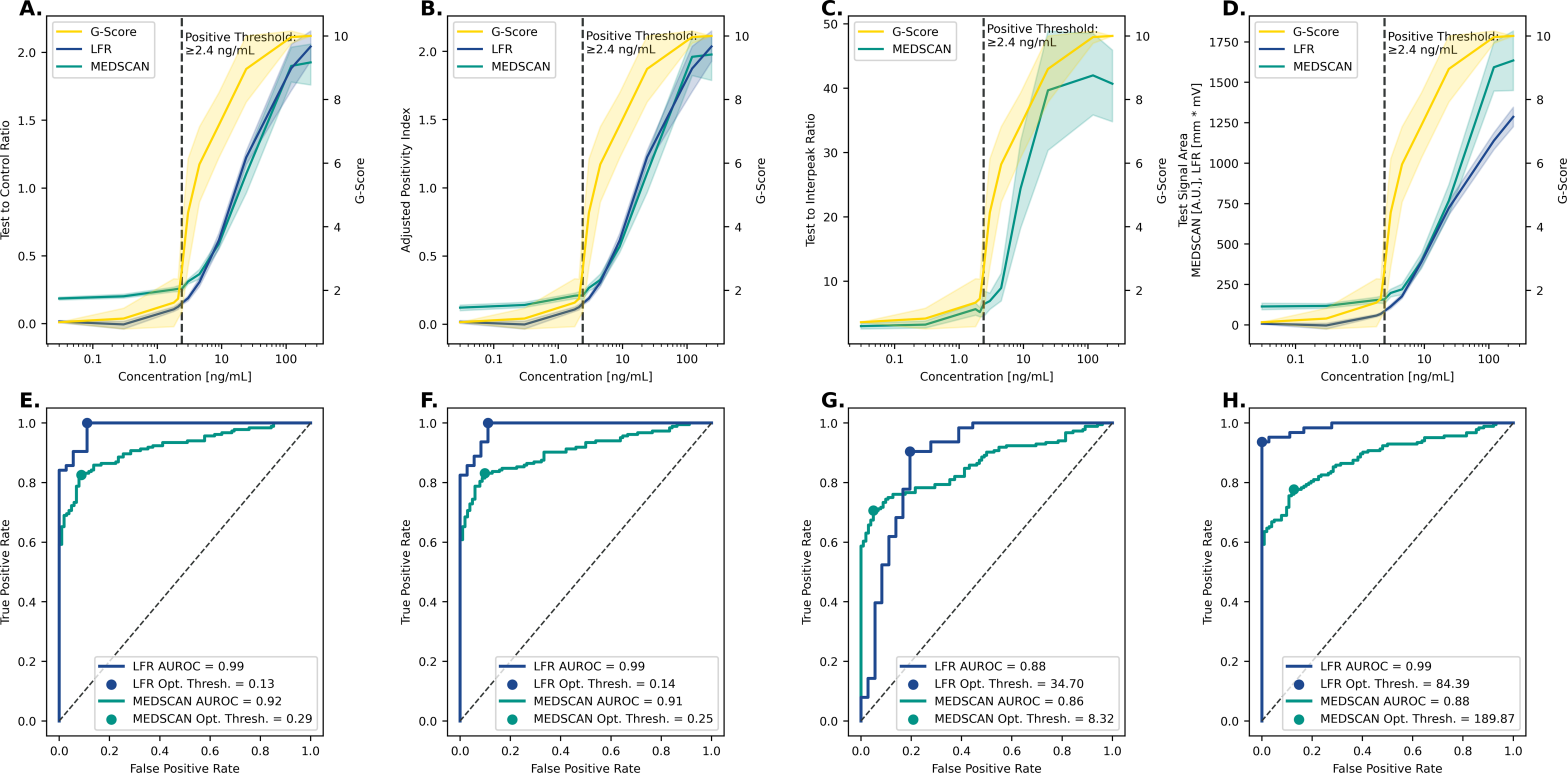
Dose–response curves (top row) and receiver operating characteristic (ROC) curves (bottom row) for four MEDSCAN signal metrics. Each column represents one metric: **(A, E)** test-to-control ratio, **(B, F)** adjusted positivity index (API), **(C, G)** test-to-interpeak ratio, and **(D, H)** test signal area. In dose–response panels, MEDSCAN (green) and LFR (blue, except panel C) values are plotted against CCA concentration, with G-Scores (yellow) overlaid on the right-hand y-axis; shaded regions indicate 95% confidence intervals for MEDSCAN and LFR, and standard deviation for G-Score. The dashed vertical line marks the established positivity threshold (≥2.4 ng/mL). In ROC panels, AUROC values and optimal cutoff thresholds are shown in the figure legends.

We next evaluated which signal representations produced stable positivity thresholds. Threshold analysis revealed that the optimal thresholds for the machine-based diagnostic metrics were robust across multiple signal representations (**Figure 5**). For the MEDSCAN Lab app, thresholds were determined independently for the raw test-line signal, the test-to-interpeak ratio, the test-to-control ratio, and the API (**Figure 5**, summarized in **Table 1**). The API yielded an optimal threshold of 0.25 (Youden’s J = 0.73; AUROC = 0.91; **Figures 5C, D**), while the test-to-control ratio threshold was 0.29 (Youden’s J = 0.74; AUROC = 0.92; **Figures 5A, B**). The test-to-interpeak ratio threshold was 8.32 (AUROC = 0.86; **Figures 5E, F**). Importantly, a threshold based solely on the test-line signal also demonstrated strong discriminative performance (Youden’s J = 0.65; AUROC = 0.88; **Figures 5G, H**), supporting use cases where the control line may be weaker but remains valid.

**Table 1:** MEDSCAN positivity thresholds based on test-line–only and normalized signal metrics.

Metric	Normalization	Threshold value	AUROC
Test line signal	None	189.87	0.88
Test-to-interpeak ratio	Background corrected	8.32	0.86
Test-to-control ratio	Control normalized	0.29	0.92
Adjusted positivity index (API)	Control-normalized + background-corrected	0.25	0.91

Optimal thresholds were determined by maximizing Youden’s J (eq. 2) against the ≥2.4 ng/mL concentration-based ground truth, pooled across all users and sites.

### Reproducibility and agreement across users, sites, and devices

To further assess reproducibility, we evaluated agreement among users, sites, and devices. At a summary level, agreement across users showed generally high consistency (**Supplementary Table 2**; **Supplementary Figure 2**), although some user pairs demonstrated lower agreement, reflecting residual variability attributable to differences in devices, environmental conditions, or individual handling. Site-level agreement was more stable: aggregated binary classifications yielded kappa values of 0.82 for KEMRI versus VU and 0.81 for LUMC versus VU, and was lower at 0.65 for KEMRI versus LUMC (**Supplementary Table 1**). ICC analyses supported these trends, with high overall inter-user reliability for both the MEDSCAN Lab app and visual G-Scores (ICC1 ≈ 0.94, **Table 2**) and excellent intra-site reliability at LUMC and KEMRI (ICC1 = 0.98 and 0.95, respectively), with slightly lower consistency at VU (ICC1 = 0.89, **Table 2**). Device-level kappas were less stable due to unequal numbers of tests contributed by different device models, with some devices represented by only a small number of observations (**Supplementary Table 3**). Pairwise kappa values between diagnostic methods ranged from 0.55 (LFR vs. MEDSCAN) to 0.75 (MEDSCAN vs. G-Score; **Supplementary Table 1**), with the lower LFR agreement reflecting the smaller sample size available at the single site where LFR data were collected. MEDSCAN signal metrics stratified by device and by user are shown in **Supplementary Figures 3**, **4**, respectively.

**Table 2:** Top: Inter-user variability across aggregated across all users at all sites, for the MEDSCAN Lab app (using API) and visual G-Scores; Bottom: Intra-site variability across sites.

Inter-user variability aggregated across all users at all sites						
Metric	ICC1	F	df1	df2	p-value	95% CI
MEDSCAN Lab app	0.94	144.69	9	80	< 0.001	[0.87, 0.98]
Visual G-Score	0.94	148.42	9	80	< 0.001	[0.88, 0.98]
Intra-site variability for the MEDSCAN Lab app						
Site	ICC1	F	df1	df2	p-value	95% CI
LUMC	0.98793	142.98	9	20	3.67×10^-^¹^6^	[0.94, 0.99]
KEMRI	0.9450	52.53	9	20	5.82×10^-^¹²	[0.85, 0.98]
VU	0.8948	26.50	9	20	3.25×10^-9^	[0.74, 0.97]

To provide a direct comparison between MEDSCAN and visual interpretation, MEDSCAN API values were plotted against visual G-Scores for all paired observations (**Supplementary Figure 5**). Binary agreement between the two methods was 87.1% (249/286): both methods classified positive in 137 cases (47.9%) and both negative in 112 cases (39.2%). In 25 discordant cases (8.7%), MEDSCAN classified positive while the visual read was negative; in 12 cases (4.2%), the reverse occurred.

## Discussion

This multi-site laboratory evaluation demonstrated that the MEDSCAN Lab app provides reliable, reproducible interpretation of the POC-CCA3 test across different users, environments, and smartphones. As expected, the MEDSCAN Lab app, visual G-Score interpretation, and the benchtop LFR each produced a clear dose–response relationship, aligning with findings from earlier work on standardizing interpretation of POC-CCA results (Casacuberta et al., 2016; Casacuberta-Partal et al., 2019; Mewamba et al., 2021; Carvalho et al., 2024). Threshold analyses further confirmed that both the MEDSCAN Lab app and the LFR effectively discriminated positive from negative samples at the manufacturer’s targeted concentration cut-off, yielding high AUROC values comparable to prior digital and reader-based evaluations of POC-CCA (Casacuberta et al., 2016; Mewamba et al., 2021). Sensitivity and specificity at each optimal operating point are reported in **Supplementary Table 4**. These values reflect the specific concentration distribution tested in this laboratory evaluation and should not be interpreted as estimates of clinical sensitivity or specificity, which will depend on the distribution of infection intensities encountered in the field. Recent work has similarly emphasized the importance of evaluating diagnostic operating points against programmatic specificity requirements for schistosomiasis surveillance (Díaz de León Derby et al., 2025).

Multiple quantitative metrics—including raw test signal, test-to-control ratio, test-to-interpeak ratio, and the adjusted positivity index (**Table 1**)—showed consistent dose-response behavior across CCA concentrations. Notably, the test-line–only threshold demonstrated strong discriminative performance, supporting a control-independent operational mode that aligns with how POC-CCA tests are currently interpreted in practice and providing a straightforward starting point for field implementation. Ratio-based metrics offer additional analytical value by normalizing for variation in image capture conditions, analogous to approaches used in previous image-analysis studies (Moore et al., 2021); however, both the control line and the interpeak signal may behave differently in endemic urine samples, where matrix effects could introduce background staining or alter signal baselines. Because the control line on the POC-CCA3 is not calibrated and is included only to confirm test validity, the robustness of ratio-based normalization beyond the present laboratory setting will need to be confirmed through field evaluation. Future work may explore allowing users to select among metrics depending on field conditions or POC-CCA3 batch characteristics. Additionally, the spiked urine samples used in this study were prepared from healthy human control urine spiked with AWA-TCA, which performs equivalently to native CCA in immunochemical assays including ELISA and lateral flow formats. However, clinical specimens from endemic areas may exhibit additional matrix variability (e.g., pH, specific gravity, endogenous substances) not captured by this laboratory evaluation.

Within this laboratory evaluation, these quantitative metrics primarily influenced the gradation between strong and moderate positives rather than the binary classification of positive and negative samples, suggesting that device-to-device or environmental variation had minimal impact on diagnostic outcomes. This observation is relevant to the long-standing challenge of interpreting visually ambiguous “trace” POC-CCA results. For the machine-based methods (MEDSCAN and LFR), the concentration-based positivity threshold of ≥2.4 ng/mL (Polman et al., 2000) was used for ROC analysis. For visual interpretation, a conservative G-Score ≥4 cutoff was applied for binarization, which excludes faint “trace” readings (typically G2–G3). The classification of trace results remains an active area of discussion: earlier POC-CCA batches supported interpreting any visible line as positive, but increased variability in later batches has complicated this recommendation (Clark et al., 2022). Other studies have proposed G≥3 as an optimal cutoff for positivity (Casacuberta-Partal et al., 2019). Over multiple production batches of the POC-CCA, G-Score 4 has generally corresponded to a CCA concentration of approximately 2.4 ng/mL; however, the G-Score is a semi-quantitative visual interpretation of the assay signal rather than a calibrated measurement, and the two thresholds reflect the same analytical boundary from different measurement perspectives. Although the present study did not explicitly evaluate trace categories, MEDSCAN’s quantitative, continuous signal measurements provide a standardized and reproducible representation of test intensity that could support future, data-driven evaluation of borderline results, without relying solely on subjective visual interpretation.

ICC analyses demonstrated high inter-user and intra-site reliability, and complementary Cohen’s kappa analyses confirmed strong binary agreement for most user pairs, consistent with findings from other controlled methodological evaluations of POC-CCA interpretation (Casacuberta et al., 2016; Carvalho et al., 2024). Residual variability likely reflects environmental influences (e.g., lighting), natural differences in smartphone auto-exposure processing, and individual handling differences—factors that have been noted as challenges in other mobile-health diagnostic systems (Wood et al., 2019; Greve et al., 2022). The use of ratio-based metrics (test-to-control, test-to-interpeak, API) provides inherent normalization against global illumination variation, and the inter-site ICC results (0.89–0.98) suggest that the algorithm tolerates the lighting differences encountered across our three laboratory environments. More extreme lighting variation in field settings may pose additional challenges, which the ongoing field study will evaluate. Device-level comparisons were more variable. Importantly, phone models were not shared across sites, meaning that device and site effects are fully confounded in this study. Cross-site threshold validation using the API metric showed that KEMRI and LUMC derived nearly identical thresholds (0.252 and 0.262, respectively) that cross-validated well (KEMRI→LUMC: Se=95%, Sp=92%). VU derived a higher threshold (0.375), likely reflecting make- and model-specific differences in non-linear image processing (e.g., HDR tone mapping) that ratio-based normalization does not fully correct (**Supplementary Figure 6**). The ongoing field study, which uses overlapping device sets across sites, will help disentangle device and site effects. As smartphone camera hardware and OS-level image processing (e.g., auto-exposure and tone mapping) can introduce systematic shifts in apparent intensity that are outside the application’s control, normalization strategies—using the control line and/or interpeak background—can help stabilize interpretation and improve comparability across devices. Nonetheless, the overall pattern—positive agreement across a heterogeneous set of smartphones—suggests that the MEDSCAN image-analysis algorithm is robust to typical differences in consumer hardware, consistent with findings from mobile-based malaria and HIV test readers (Scherr et al., 2016; Moore et al., 2021; Schnall et al., 2025) and recent AI-driven diagnostic systems which have likewise demonstrated high concordance with expert readers (Bahati et al., 2025). Unlike prior image-analysis or reader-based approaches that have remained largely confined to research settings, MEDSCAN integrates automated interpretation directly into a field-deployable digital surveillance platform, coupling diagnostic standardization with real-time data capture and programmatic analytics.

The LFR provided highly precise measurements, with enhanced resolution at low CCA concentrations. In this evaluation, its primary role was to validate the trends observed with the MEDSCAN Lab app. The close correspondence between MEDSCAN and LFR dose–response patterns confirms that the app captures the same underlying analytical behavior detectable with a calibrated benchtop instrument. Bland–Altman analysis of the adjusted positivity index (API) between MEDSCAN and LFR revealed negligible mean bias (0.01) with 95% limits of agreement of −0.53 to 0.54; residuals plotted against concentration showed close agreement at low-to-moderate concentrations, with increased variability and occasional MEDSCAN underestimation at the highest analyte levels (**Supplementary Figure 7**). When ROC analysis is restricted to the low-to-moderate concentration range (0–4.5 ng/mL), MEDSCAN AUROC values decrease by 0.09–0.15 (**SI Figure 8**), reflecting the removal of clinically relevant concentrations that are detected with near-certainty rather than a correction for inflated performance. Near the limit of detection, the more clinically meaningful comparison is between MEDSCAN and the visual interpretation it replaces, where the API vs. G-Score analysis demonstrates 87% agreement, with MEDSCAN detecting more borderline positives than visual reads missed in the reverse direction (**Supplementary Figure 5**). While the present study focused on binary positivity outcomes aligned with the established concentration threshold of ≥2.4 ng/mL (Polman et al., 2000; van Dam et al., 2004), finer distinctions in signal intensity could be relevant for applications such as drug-efficacy monitoring, estimating worm burden, or differentiating light versus heavy infections in persistent transmission hotspots. MEDSCAN generates quantitative signal outputs that represent a valuable feature of the platform and should be explored further for applications beyond binary diagnostic classification.

The non-zero MEDSCAN test signal observed at 0 ng/mL (**Figure 4B**) likely reflects the sensitivity of broadband smartphone imaging to the physical properties of the test membrane: capture antibodies striped onto the nitrocellulose during manufacturing create a localized change in membrane opacity detectable by a broadband camera sensor, even in the absence of colorimetric signal development. By contrast, the LFR uses narrowband LED illumination tuned to the gold nanoparticle absorption wavelength, at which the dried protein stripe is effectively transparent—consistent with the near-zero LFR test signal at 0 ng/mL (**Figure 4C**). This baseline offset does not affect diagnostic classification, as the ROC-derived thresholds (**Table 1**) are set above this non-specific optical background.

Together, these findings demonstrate that MEDSCAN provides accurate, consistent, and scalable interpretation of the POC-CCA test, offering a practical alternative to visual scoring and a standardized complement to benchtop LFRs. For field deployment, these results support either a test-line–only threshold of 189.87 or a normalized threshold using the test-to-control ratio (0.29) or API (0.25). Together, these analyses define operational positivity thresholds for MEDSCAN—based on test-line signal alone or normalized metrics—that can be applied directly in field surveillance workflows. While continued refinement of image-based approaches—particularly moving from a controlled laboratory setting to more varied field and sample conditions—will further strengthen performance, the MEDSCAN platform already enables value beyond test interpretation alone. Integration of automated results with geolocation, timestamping, and user metadata creates opportunities for enhanced, real-time surveillance analytics to support schistosomiasis control programs as they push towards elimination.

### Limitations

This study has several limitations. First, the visual scoring of the POC-CCA tests using the G-Score system was not blinded, meaning that raters were aware of sample concentrations, which may have introduced bias in the scoring. Additionally, the intra-site variability analyses (ICC) were based on a relatively small sample size (n=3 users per site), potentially limiting the generalizability of the observed inter-rater variability. Finally, phone models were not shared across sites, meaning that device and site effects cannot be independently separated in this study.

### Future directions

Future work will focus on refining specific algorithmic and operational parameters of the MEDSCAN platform, including image preprocessing, signal extraction windows, and decision thresholds, to optimize performance for different surveillance and programmatic use cases. While the present study evaluated a single production batch of the POC-CCA3 under controlled laboratory conditions, future work will prioritize validating MEDSCAN performance across diverse field conditions rather than conducting additional laboratory-only evaluations.

Because the control line of the POC-CCA test is not calibrated during manufacturing and may vary with sample composition, lighting, or test handling, alternative normalization strategies warrant further investigation. Approaches that reduce reliance on the control line—such as normalization based on the interpeak (background) signal, use of raw test-line intensity, incorporation of device metadata (e.g., camera exposure or luminance estimates), or other image-based corrections—may improve robustness when tests are used in heterogeneous, real-world environments.

Importantly, a large-scale observational field study is currently underway to evaluate the performance of the MEDSCAN application under routine POC-CCA surveillance conditions. This study will assess not only diagnostic agreement between visual G-Scores and quantitative MEDSCAN outcomes but also the added value of MEDSCAN’s integrated digital features—including geolocation, timestamping, user audit trails, and centralized data aggregation—for supporting real-time surveillance and programmatic decision-making in endemic settings. Together, these efforts will clarify how MEDSCAN can best support schistosomiasis control and surveillance beyond controlled laboratory evaluations.

## Conclusion

Our evaluation confirms that the MEDSCAN Lab app reliably replicates and often improves upon visual interpretation (G-Scores) of the POC-CCA for schistosomiasis diagnostics, providing objective, consistent results across multiple users, international testing sites, and smartphones. While some variability related to smartphone hardware underscores the importance of device standardization or improved normalization techniques, MEDSCAN’s scalability makes it highly suitable for field-based applications. Further optimization and standardization will enhance MEDSCAN’s diagnostic accuracy and reliability, significantly contributing to improved surveillance and control strategies for schistosomiasis in resource-limited settings.

## Data Availability

The datasets presented in this study can be found in online repositories. The names of the repository/repositories and accession number(s) can be found below: https://doi.org/10.6084/m9.figshare.31838509.
