# Supplementary material for: A multi-site laboratory evaluation of the MEDSCAN application for automated POC-CCA interpretation
Source: Front Parasitol. 2026 Jun 19;5:1837310. doi: 10.3389/fpara.2026.1837310 (PMC13329796; doi:10.3389/fpara.2026.1837310)
Supplement: Supplementary file 1 [file DataSheet1.docx]

# Supporting Information

## Details of Training Computer Vision Algorithm

The MEDSCAN Lab App uses an image processing algorithm (MEDSCAN RDT Reader, v 2.0.2) designed to analyze lateral flow assay results. At present, the software is trained on POC-CCA3 tests, but our previous work developing algorithms for interpretation of diagnostic tests for malaria, HIV, and COVID-19 is expected to be integrated shortly. The computer vision pipeline can be separated into two major components, each specific to an image processing task: 1) recognition and isolation of the regions of interest of a diagnostic test (i.e., cassette, sample well, and test membrane) within a photograph, with varying orientations, angles of incidence, and background composition, and 2) analysis of the region of interest that contains the test outcome (i.e., the test membrane) to determine test results.

To find and isolate the cassette (and other test regions of interest) from a photograph, a maskrcnn network was trained from 271 photographs of the POC-CCA3 test. The photographs were annotated using VGG Image Annotator software (VIA; Visual Geometry Group, Oxford University). Training occurred on an iMac 2017 (iMac Pro, 3.2 GHz 8-core Intel Xeon W, 32 GB 2666 MHz DDR4). Training was completed in several hours.

Using the trained model, inference is performed on a photograph to identify the cassette and other regions of interest, the photograph is rotated, using the locations of the ROIs, in such a way that the membrane is horizontally aligned and the control line always on the right side.

Image pre-processing steps are performed next to condition the image for signal analysis. From here, a linescan is performed over a portion of the cropped image. Pixel intensities are averaged over the transverse direction, resulting in a single averaged pixel intensity per positional coordinate of the linescan. A peak-finding algorithm searches for a control line in the downstream half of the linescan; if it cannot find one above a given threshold, the test is classified as invalid. Our algorithm uses a combination of grayscale and color linescans to enhance accuracy. After finding the control line on valid tests, it uses a guess-and-search approach to find the test line peak around its expected location. The peaks (control and test) are numerically integrated with the trapezoidal rule to a given half-width of each side of the peak maxima; the baselines are subtracted from the peak area. The resulting signals are then used in different metrics to determine positivity.

Evaluation of the MEDSCAN Lab App can be separated into two aspects: cassette detection and identification of the test membrane’s viewing window from a photograph, and signal processing of the test membrane to interpret test results. The latter is described in detail in the body of the manuscript, and the former described in more detail below.

# mAP vs IoU Discussion

Mean average precision (mAP) against increasing Intersection of Union (IoU) thresholds were evaluated for overall detections, as well as for the individual regions—cassette, membrane, and sample well—and the results are shown in **SI Figure 9**. At a high-level, this shows how well the bounding boxes of each region predicted by the computer vision model overlap with the region annotations that were used in training. The overall mAP remains relatively high until an IoU threshold of about 0.85, at which point a drop-off occurs; among the regions, the membrane retains better performance, while the well exhibits a steeper decline. Overall and region-specific detections were all satisfactory, as no errors were noted in the extraction of these regions from a photo, and the linescan functionality of the algorithm was able to find a control peak in all photos.

While identification of the cassette exterior and well play an important role in re-orientation of the photograph for the evaluation of the test membrane, having higher performance tighter tolerances for the membrane is the most important. If these tolerances were not at a performant level, the linescan algorithm that relies on peak finding in specific regions would fail. In other words: if the detected membrane is skewed (either a result of the rotation being off from the cassette and well detection, or from the membrane detection being inaccurate), the algorithm won’t find a control line where it expects to. In practice, if the algorithm cannot detect the cassette with sufficient confidence, the app requests that the user retake the photograph; if the linescan subsequently fails to identify a control peak, the test is classified as invalid. While retake requests did occur during this evaluation, no undetectable control line errors were recorded. The mAP metric characterizes detection precision, while the clinically relevant safeguard is the pass/fail gate at the linescan stage. We would like to note here that proper error handling is important, as we anticipate usage where photographs might not be conducive to object detection. Gracefully handling any errors and alerting users in the app with guidance to continue is critical.

## Supplemental Figures


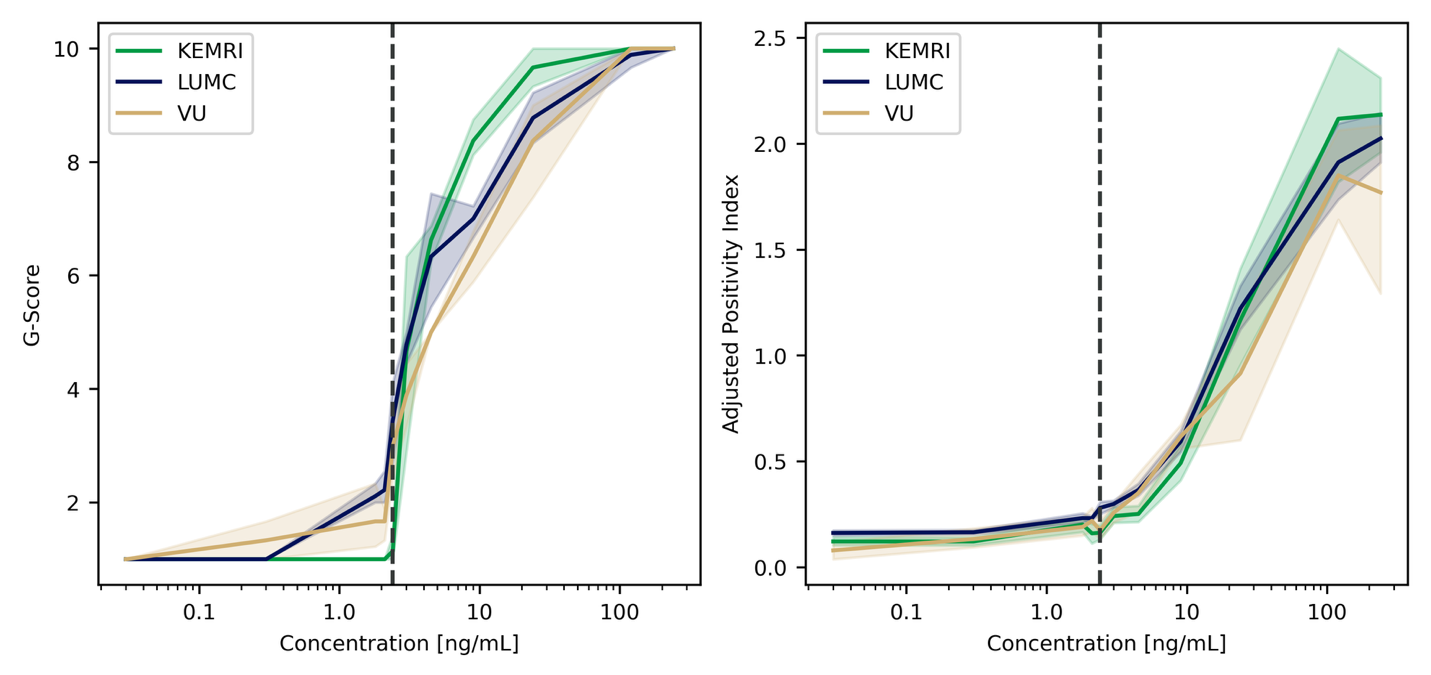


**SI FIGURE 1.** Aggregations of all users by site, across all concentrations, for **A.** G-Scores, and **B.** Adjusted Positivity Index.


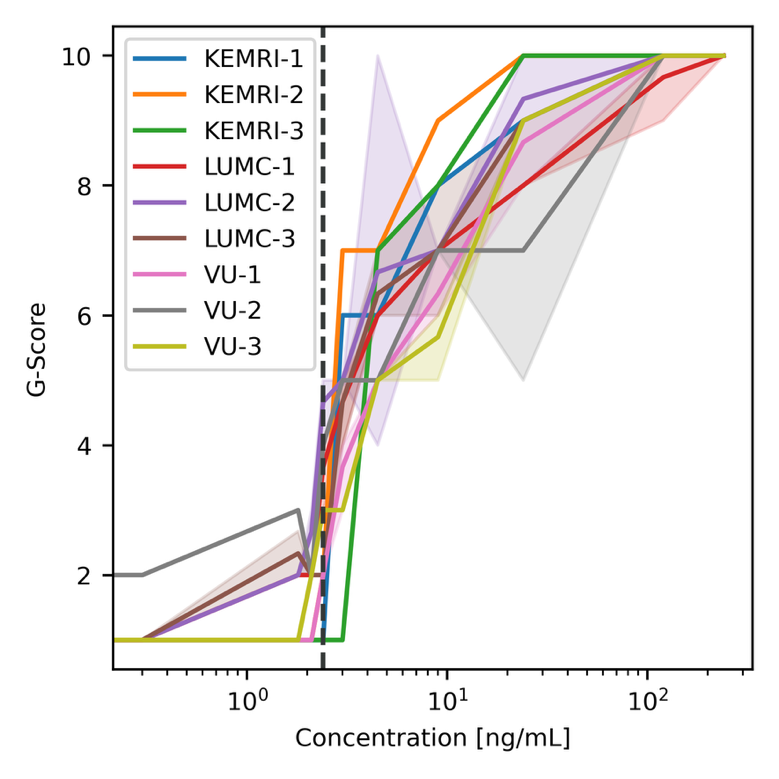


**SI FIGURE 2.** G Scores across all concentrations, grouped by user.


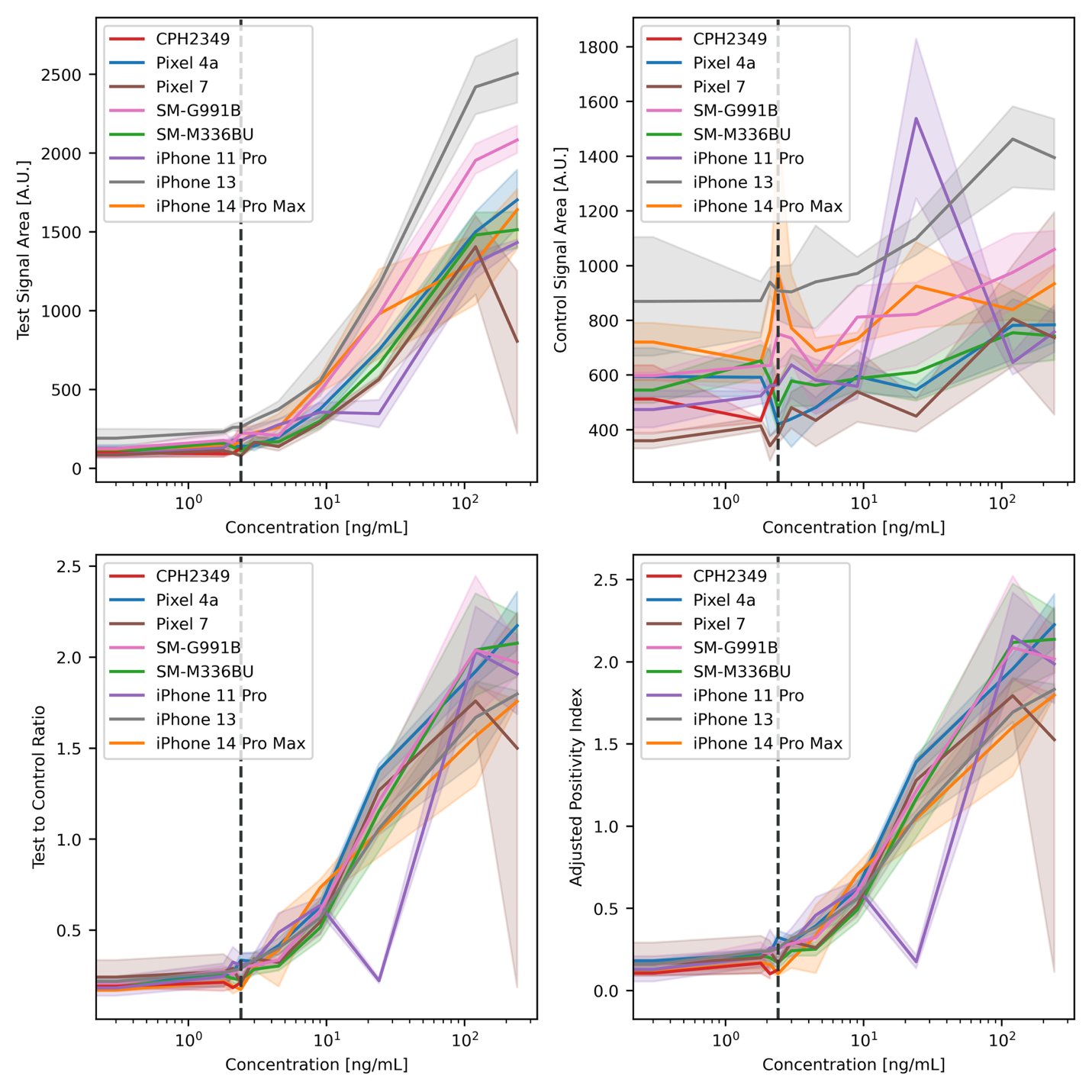


**SI FIGURE 3.** MEDSCAN signal metrics stratified by smartphone device. Mean signal measurements across eight device types used by nine users (two users both used an iPhone 13 device). **(A)** Test signal area, **(B)** control signal area, **(C)** test-to-control ratio, and **(D)** adjusted positivity index plotted against analyte concentration. Dashed line indicates 2.4 ng/mL positivity threshold. Shaded regions show 95% confidence intervals.


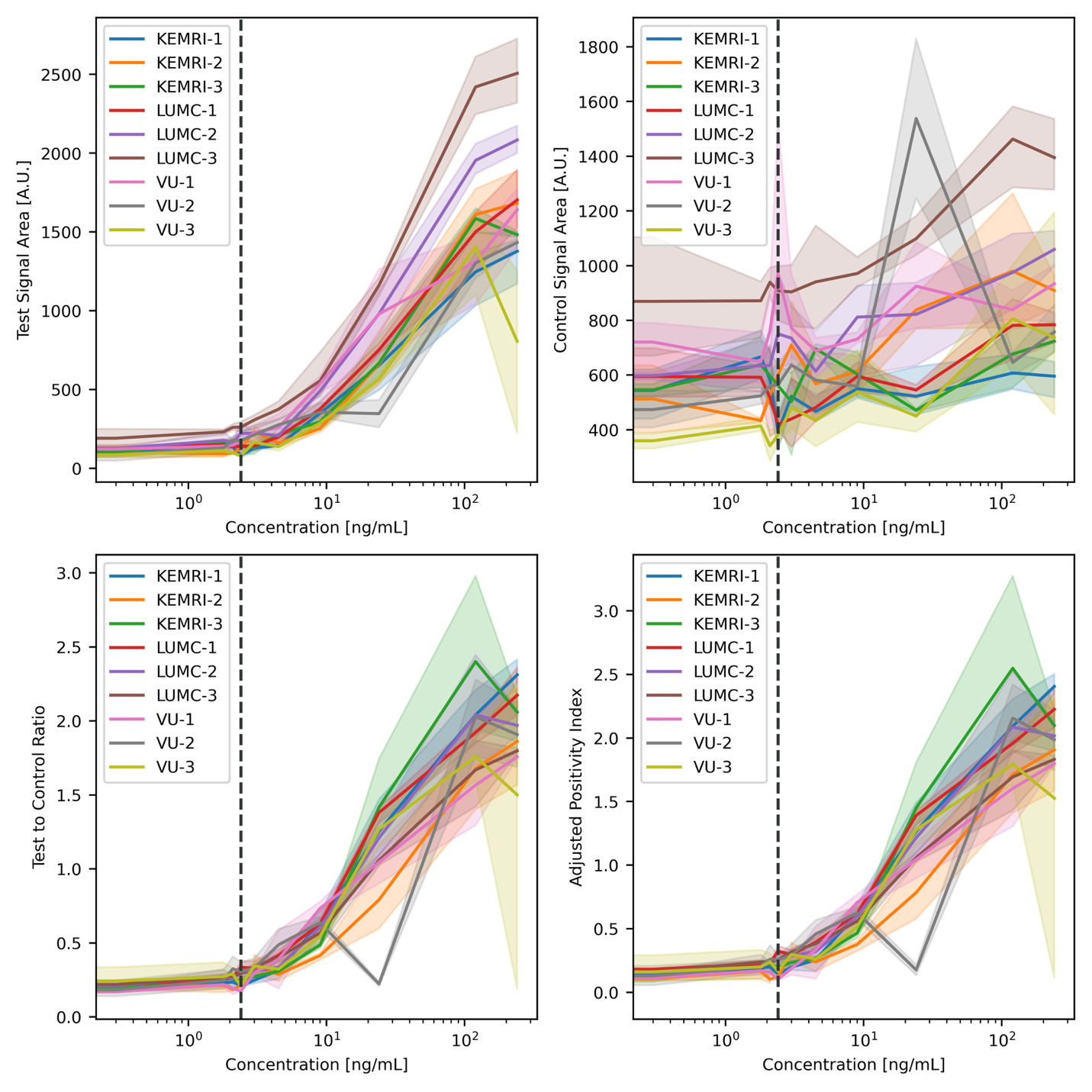


**SI FIGURE 4.** MEDSCAN signal metrics stratified by user. Mean signal measurements across eight device types used by nine users (two users both used an iPhone 13 device). **(A)** Test signal area, **(B)** control signal area, **(C)** test-to-control ratio, and **(D)** adjusted positivity index plotted against analyte concentration. Dashed line indicates 2.4 ng/mL positivity threshold. Shaded regions show 95% confidence intervals.

**
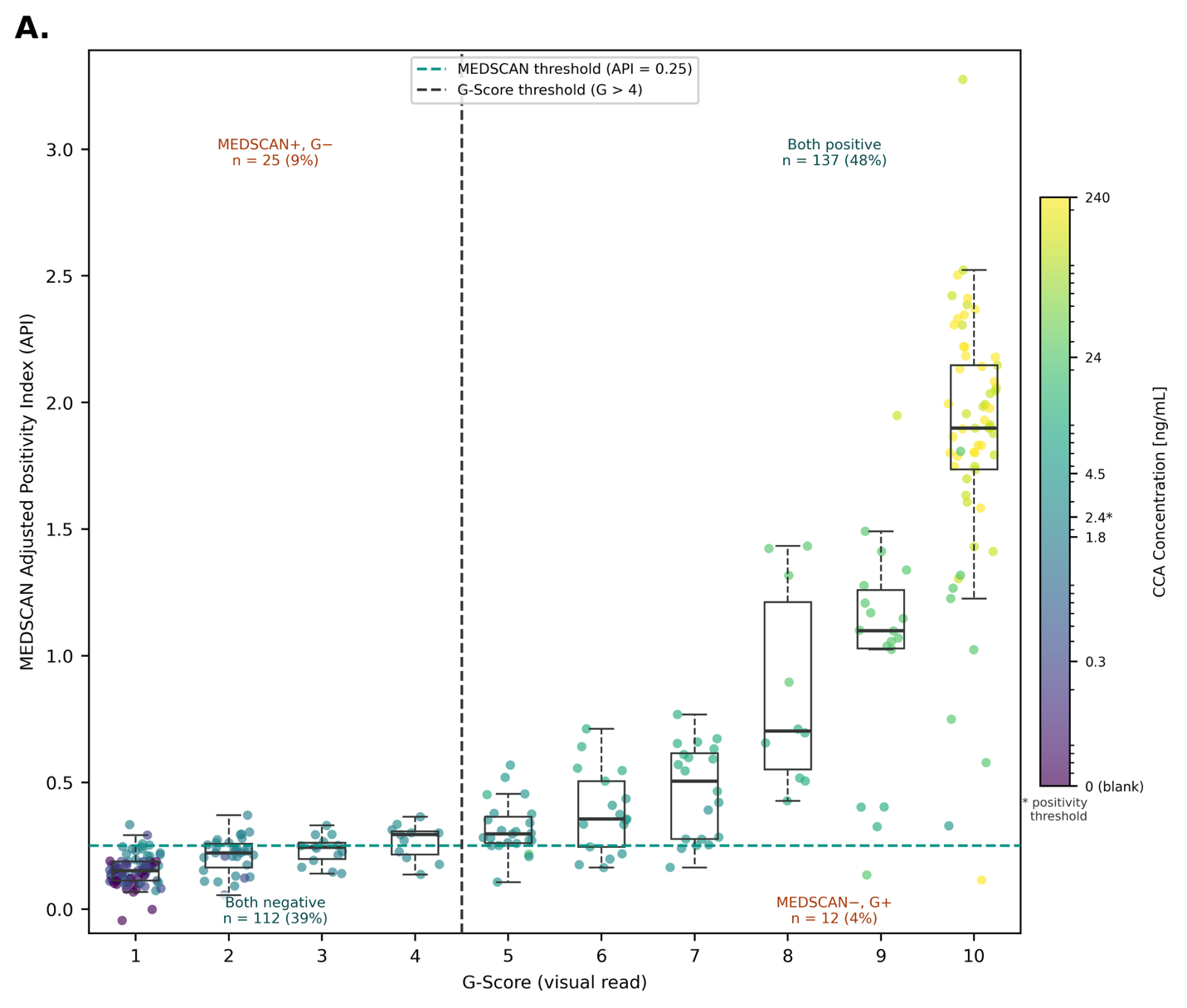
**

**SI FIGURE 5.** MEDSCAN Adjusted Positivity Index (API) versus visual G-Score for all 286 paired observations. Points are colored by CCA concentration (ng/mL) and jittered horizontally for visibility. Box plots show the median and interquartile range per G-Score level. Dashed lines indicate the MEDSCAN positivity threshold (API = 0.25, horizontal) and visual positivity threshold (G-Score ≥ 4, vertical). Quadrant labels show concordance: both positive (n=137, 48%), both negative (n=112, 39%), MEDSCAN+/G− (n=25, 9%), and MEDSCAN−/G+ (n=12, 4%).

**
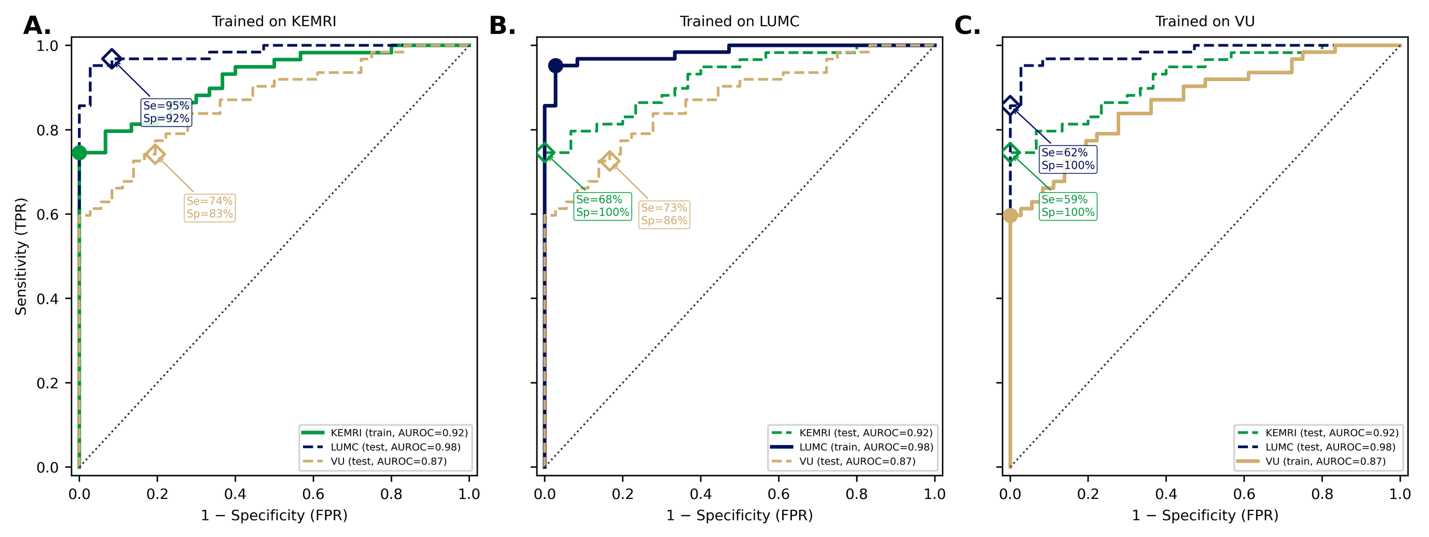
**

**SI FIGURE 6.** Cross-site threshold validation for the MEDSCAN Adjusted Positivity Index (API). Each panel shows ROC curves when the optimal threshold is derived from one site (solid line, training) and applied to the other two sites (dashed lines, test). A: Trained on KEMRI. B: Trained on LUMC. C: Trained on VU. Sensitivity and specificity at each cross-applied threshold are annotated.


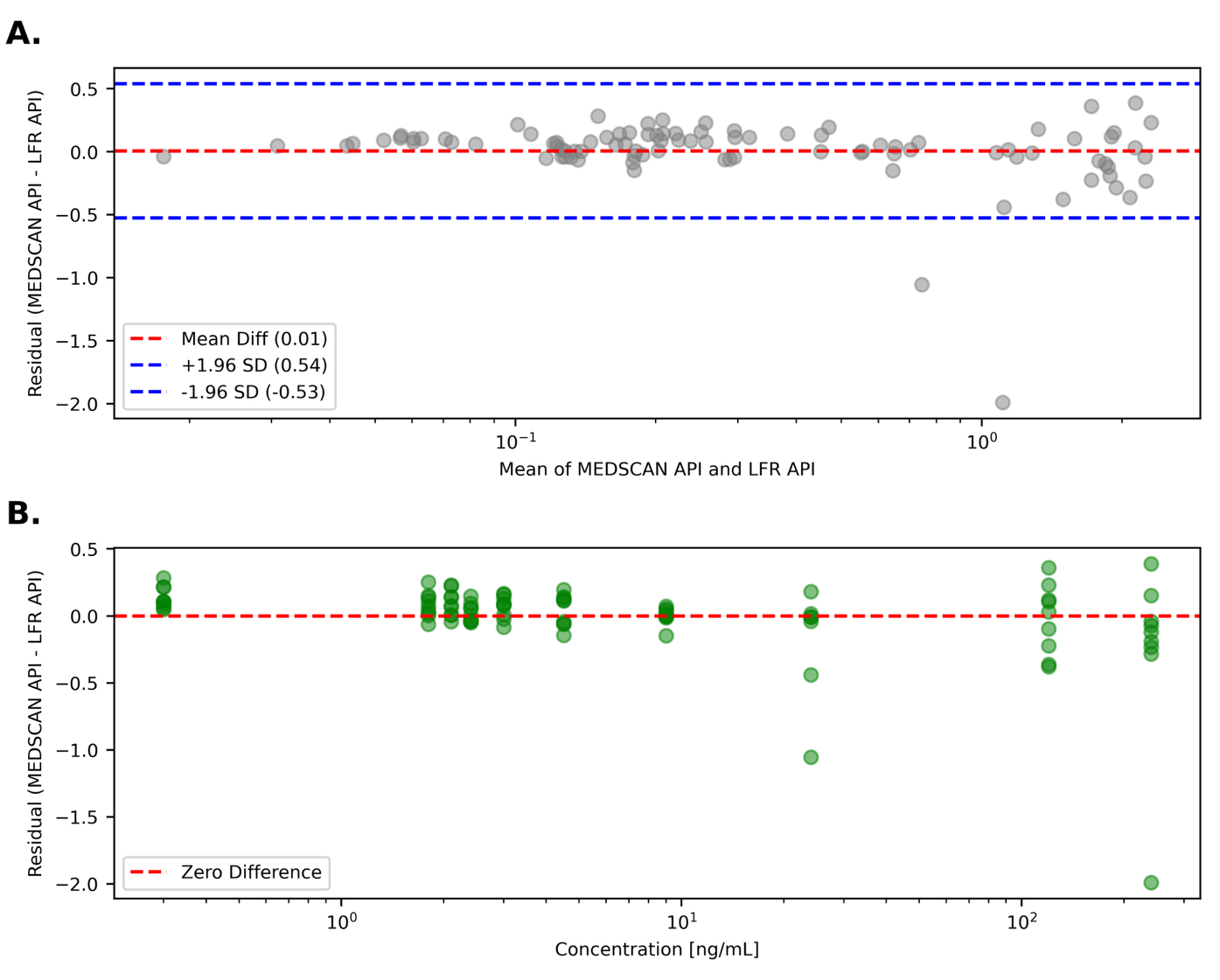


**SI FIGURE 7.** **A.** Bland–Altman plot of the adjusted positivity index (API): difference between MEDSCAN API and LFR API versus their mean. **B.** Residual plot: difference between MEDSCAN API and LFR API versus analyte concentration (ng CCA/mL). Dashed lines indicate the mean difference and 95% limits of agreement.

**
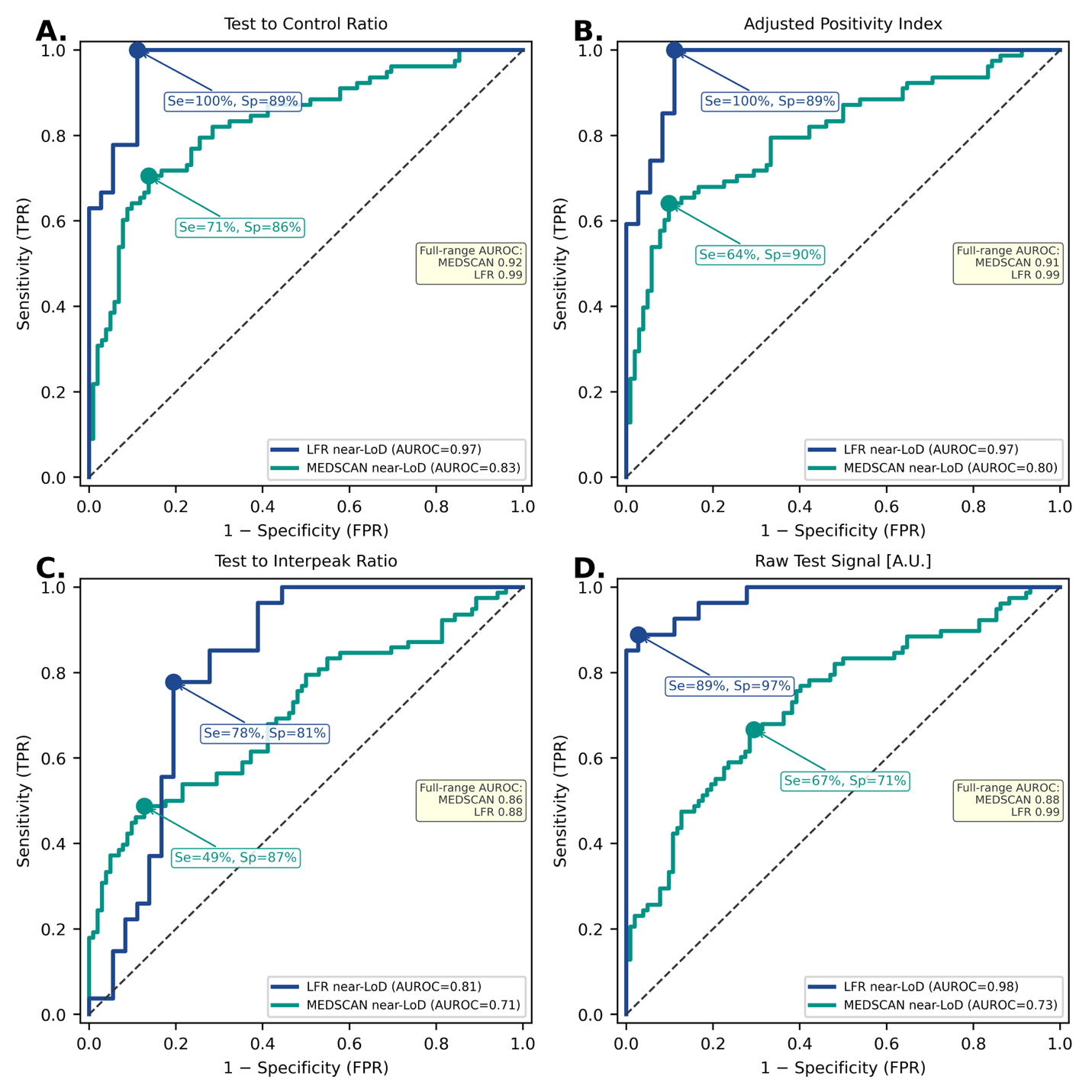
**

**SI FIGURE 8.** ROC curves for MEDSCAN and LFR signal metrics restricted to the low-to-moderate concentration range (0–4.5 ng/mL), excluding the 24 and 240 ng/mL samples. AUROC values and optimal thresholds are shown in figure legends. Compared to the full-range analysis (Figure 5), MEDSCAN AUROC values decrease by 0.09–0.15, reflecting the inherent difficulty of discriminating near-threshold concentrations using broadband smartphone imaging.


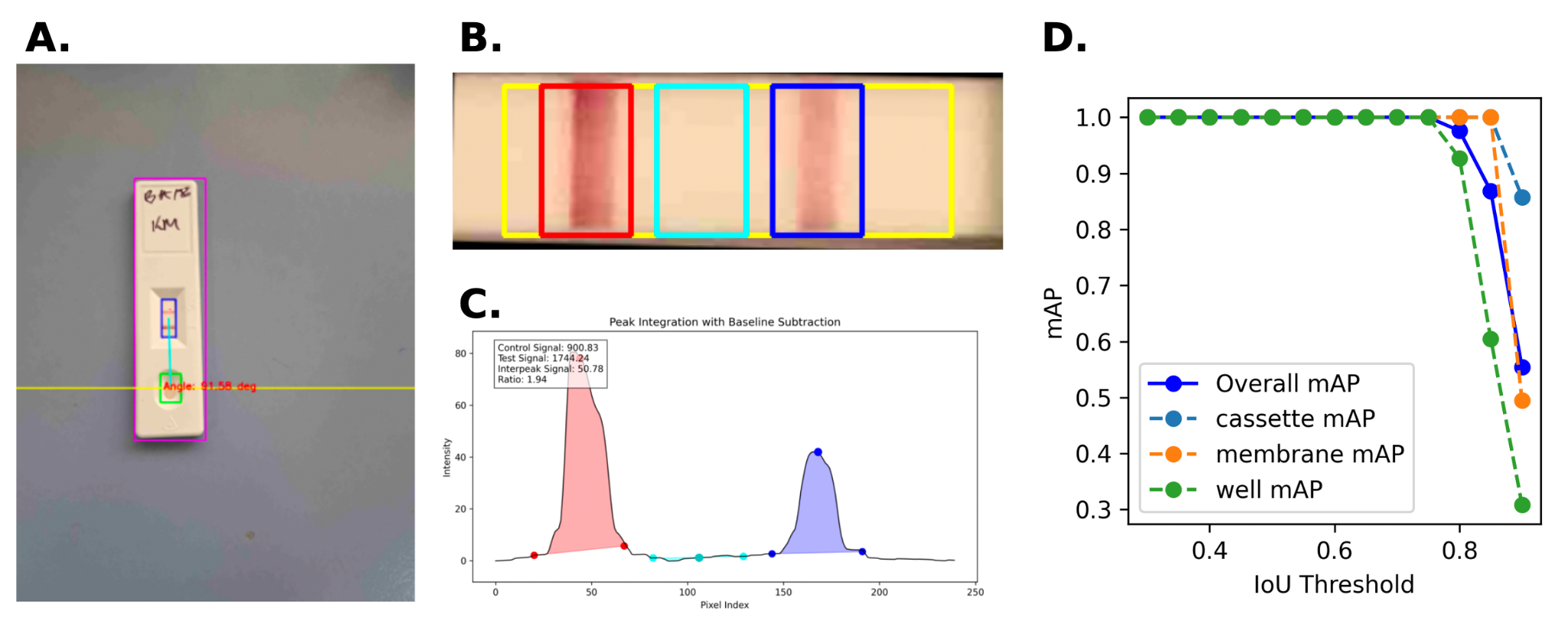


**SI FIGURE 9.** **A-C.** Example computer vision steps in the MEDSCAN algorithm: **A.** cassette detection, **B.** test and control line finding, **C.** linescan analysis and signal integration. **D.** Mean average Precision (mAP) against varying Intersection of Union thresholds for all and individual regions of interest.

**SI TABLE 1. (Top) Pairwise site Cohen’s kappa comparisons for MEDSCAN. (Bottom) Cohen’s kappa values comparing methods.**

| **MEDSCAN pairwise Cohen's Kappa across sites** | | |
| --- | --- | --- |
| Site 1 | Site 2 | Cohen’s kappa |
| KEMRI | LUMC | 0.645 |
| KEMRI | VU | 0.820 |
| LUMC | VU | 0.813 |
| **Aggregate method comparisons** | | |
| Method 1 | Method 2 | Cohen’s kappa |
| MEDSCAN Lab App | G-Score | 0.75 |
| LFR | MEDSCAN Lab App | 0.55 |
| LFR | G-Score | 0.71 |

**SI TABLE 2.** Pairwise Cohen's Kappa across users.

| Group1 | Group2 | CohenKappa |
| --- | --- | --- |
| KEMRI-1 | KEMRI-2 | 0.63 |
| KEMRI-1 | KEMRI-3 | 1.000 |
| KEMRI-1 | LUMC-1 | 0.65 |
| KEMRI-1 | LUMC-2 | 0.65 |
| KEMRI-1 | LUMC-3 | 0.65 |
| KEMRI-1 | VU-1 | 1.00 |
| KEMRI-1 | VU-2 | 0.29 |
| KEMRI-1 | VU-3 | 0.82 |
| KEMRI-2 | KEMRI-3 | 0.60 |
| KEMRI-2 | LUMC-1 | 0.65 |
| KEMRI-2 | LUMC-2 | 0.65 |
| KEMRI-2 | LUMC-3 | 0.65 |
| KEMRI-2 | VU-1 | 0.63 |
| KEMRI-2 | VU-2 | 0.29 |
| KEMRI-2 | VU-3 | 0.82 |
| KEMRI-3 | LUMC-1 | 0.60 |
| KEMRI-3 | LUMC-2 | 0.60 |
| KEMRI-3 | LUMC-3 | 0.60 |
| KEMRI-3 | VU-1 | 1.00 |
| KEMRI-3 | VU-2 | 0.40 |
| KEMRI-3 | VU-3 | 0.80 |
| LUMC-1 | LUMC-2 | 1.00 |
| LUMC-1 | LUMC-3 | 1.00 |
| LUMC-1 | VU-1 | 0.65 |
| LUMC-1 | VU-2 | 0.61 |
| LUMC-1 | VU-3 | 0.81 |
| LUMC-2 | LUMC-3 | 1.00 |
| LUMC-2 | VU-1 | 0.65 |
| LUMC-2 | VU-2 | 0.61 |
| LUMC-2 | VU-3 | 0.81 |
| LUMC-3 | VU-1 | 0.65 |
| LUMC-3 | VU-2 | 0.61 |
| LUMC-3 | VU-3 | 0.81 |
| VU-1 | VU-2 | 0.29 |
| VU-1 | VU-3 | 0.82 |
| VU-2 | VU-3 | 0.44 |

**SI TABLE 3.** Pairwise Cohen's Kappa across devices: (excluded device CPH2349 because all Kappa values were 0 or NaN).

| Group1 | Group2 | Cohen’s Kappa |
| --- | --- | --- |
| Pixel 4a | Pixel 7 | 0.81 |
| Pixel 4a | SM-G991B | 1.00 |
| Pixel 4a | SM-M336BU | 0.65 |
| Pixel 4a | iPhone 11 Pro | 0.61 |
| Pixel 4a | iPhone 13 | 1.00 |
| Pixel 4a | iPhone 14 Pro Max | 0.65 |
| Pixel 7 | SM-G991B | 0.81 |
| Pixel 7 | SM-M336BU | 0.82 |
| Pixel 7 | iPhone 11 Pro | 0.44 |
| Pixel 7 | iPhone 13 | 0.81 |
| Pixel 7 | iPhone 14 Pro Max | 0.82 |
| SM-G991B | SM-M336BU | 0.65 |
| SM-G991B | iPhone 11 Pro | 0.61 |
| SM-G991B | iPhone 13 | 1.00 |
| SM-G991B | iPhone 14 Pro Max | 0.65 |
| SM-M336BU | iPhone 11 Pro | 0.29 |
| SM-M336BU | iPhone 13 | 0.65 |
| SM-M336BU | iPhone 14 Pro Max | 1.00 |
| iPhone 11 Pro | iPhone 13 | 0.61 |
| iPhone 11 Pro | iPhone 14 Pro Max | 0.29 |
| iPhone 13 | iPhone 14 Pro Max | 0.65 |

**SI TABLE 4.** Sensitivity and specificity at optimal Youden's J thresholds for MEDSCAN and LFR signal metrics. Ground truth: ≥2.4 ng/mL = positive. Values reflect the specific concentration distribution tested in this laboratory evaluation (0–240 ng CCA/mL in spiked urine) and should not be interpreted as estimates of clinical sensitivity or specificity.

| Method | Metric | Threshold | AUROC | Sensitivity | Specificity |
| --- | --- | --- | --- | --- | --- |
| MEDSCAN | Test-to-control ratio | 0.29 | 0.92 | 82.1% | 91.2% |
| MEDSCAN | Adjusted positivity index | 0.25 | 0.91 | 83.2% | 90.2% |
| MEDSCAN | Raw test signal | 189.87 | 0.88 | 77.2% | 87.3% |
| MEDSCAN | Test-to-interpeak ratio | 8.32 | 0.86 | 70.1% | 95.1% |
| LFR | Test-to-control ratio | 0.13 | 0.99 | 98.4% | 88.9% |
| LFR | Adjusted positivity index | 0.14 | 0.99 | 98.4% | 88.9% |
| LFR | Raw test signal | 84.39 | 0.99 | 92.1% | 100% |
| LFR | Test-to-interpeak ratio | 34.70 | 0.88 | 90.5% | 80.6% |
